# Whole-Blood Gene Expression and Hypoxic-Ischemic Encephalopathy at Birth

**DOI:** 10.1001/jamanetworkopen.2026.29378

**Published:** 2026-08-21

**Authors:** Paolo Montaldo, Jethro Herberg, Sahar Uppal, Simona Puzone, Grazia Cirillo, Emanuele Miraglia Del Giudice, Mario Cirillo, Elisabetta Caredda, Reema Garegrat, Stuti Pant, Anchal Dhawan, Thilipan Thaventhiran, Mia Cunliffe, Narendra Aladangady, Paul Fleming, Balamurugan Palanisami, Aung Soe, Palaniappan Sashikumar, Santosh Pattnayak, Sundeep Harigopal, Seetha Shankaran, Sudhin Thayyil

**Affiliations:** 1 Centre for Perinatal Neuroscience, Department of Brain Sciences, Imperial College London, London, United Kingdom; 2 Department of Women’s and Children’s Health and General and Specialized Surgery, University of Campania Luigi Vanvitelli, Naples, Italy; 3 Section of Paediatric Infectious Disease, Department of Infectious Disease, Imperial College London, London, United Kingdom; 4 Department of Advanced Medical and Surgical Sciences, University of Campania “Luigi Vanvitelli,” Naples, Italy; 5 Department of Neonatology, Homerton Healthcare NHS Foundation Trust, London, United Kingdom; 6 Centre for Genomics and Child Health, Blizard Institute, Queen Mary University of London, London, United Kingdom; 7 Neonatal Medicine, Liverpool Women’s NHS Foundation Trust, Liverpool, United Kingdom; 8 Oliver Fisher Neonatal Intensive Care Unit, Medway Maritime Hospital, Medway NHS Foundation Trust, Kent, United Kingdom; 9 Neonatal Medicine, Royal Victoria Infirmary, Newcastle Upon Tyne, United Kingdom; 10 Department of Neonatal-Perinatal Medicine, Wayne State University, Detroit, Michigan; 11 Department of Pediatrics, The University of Texas at Austin, Dell Children’s Hospital, Austin

## Abstract

**Importance:**

Mild hypoxic-ischemic encephalopathy (HIE) spans a broad spectrum of neurological dysfunction, making early identification challenging and leading to undertreatment or overtreatment.

**Objectives:**

To investigate whether whole-blood gene expression at birth is associated with HIE severity (mild, moderate, or severe), to characterize pathways underlying differential expression, and to evaluate temporal changes over the first 72 hours compared with matched healthy controls.

**Design, Setting, and Participants:**

This case-control study combines data from the COMET (Cooling in Mild Encephalopathy) pilot randomized clinical trial from October 31, 2019, to April 28, 2023, recruiting infants with mild HIE and data from 2 prospective observational studies (January 1, 2017, to June 1, 2019, and September 30, 2019, to September 30, 2025) enrolling infants with moderate or severe HIE and healthy control infants. The settings include 6 tertiary neonatal intensive care units in the United Kingdom and Italy, and the participants were full-term infants (gestational age, ≥36 weeks; birth weight, ≥1.8 kg) with mild, moderate, or severe HIE and healthy control infants. Data were analyzed from October 2025 to February 2026.

**Exposure:**

Whole-blood RNA next-generation sequencing.

**Main Outcomes and Measures:**

Measures were gene-level counts and pathway enrichment statistics. Primary outcomes were gene expression differences by HIE severity and the temporal evolution of transcriptomic profiles compared with controls.

**Results:**

A total of 112 full-term infants were included: 73 with HIE (40 with mild HIE, 29 with moderate HIE, and 4 with severe HIE; 19 infants with mild HIE [47.5%] and 20 infants with moderate or severe HIE [61.0%] were female) and 39 healthy control infants (23 female infants [59.0%]). The median birth weight was 3.3 kg (IQR, 3.0-3.6 kg) for infants with moderate or severe HIE, 3.3 kg (IQR, 3.1-3.5 kg) for infants with mild HIE, and 3.4 kg (IQR, 3.0-3.6 kg) for controls. The median gestational age was 40.0 weeks (IQR, 39.0-40.6 weeks) for infants with mild HIE, 39.3 weeks (IQR, 39.0-41.0 weeks) for infants with moderate HIE, and 39.4 weeks (IQR, 39.0-40.4 weeks) for infants with severe HIE. Differential expression analysis identified 981 genes in mild HIE and 622 genes in moderate or severe HIE (false discovery rate <0.05), with 37 genes (3.0%) shared. In mild HIE, the processing of capped intron-containing pre-mRNA was downregulated (*z* score, −0.68; *P* < .001), whereas in moderate or severe HIE, autoimmune thyroid disease signaling showed the highest significance (*z* score not determined; *P* < .001). By 72 hours, transcriptomic disturbances had substantially attenuated, particularly in mild HIE, with gene expression profiles converging toward control levels.

**Conclusions and Relevance:**

In this case-control study of infants with HIE, whole-blood gene expression at birth was associated with HIE severity, distinguishing infants with mild HIE from both controls and those with moderate or severe encephalopathy, supporting its role in risk stratification during the period when neuroprotective interventions remain possible and clinical diagnosis may still be challenging.

## Key Points

**Question:**

Is whole-blood gene expression at birth associated with the severity of hypoxic-ischemic encephalopathy (HIE), and how do transcriptomic patterns among patients with HIE change over the first 72 hours?

**Findings:**

In this case-control study including 73 infants with HIE and 39 healthy controls, whole-blood RNA sequencing identified distinct gene expression signatures for mild HIE, with minimal overlap with moderate or severe HIE. Moderate or severe HIE showed activation of adaptive and innate immune activation pathways, whereas mild HIE showed activation of cell-intrinsic stress responses that normalized by 72 hours.

**Meaning:**

In this case-control study of infants with HIE, whole-blood gene expression at birth could differentiate infants with mild HIE from infants with moderate or severe HIE; mild HIE preserves metabolic homeostasis, whereas moderate or severe HIE activates stress- and injury-related pathways.

## Teaser

This case-control study investigates whether whole-blood gene expression at birth is associated with hypoxic-ischemic encephalopathy severity (mild, moderate, or severe), characterizes the pathways underlying differential expression, and evaluates the temporal changes over the first 72 hours compared with matched healthy controls.

## Introduction

Hypoxic-ischemic encephalopathy (HIE) remains a major global cause of neonatal mortality and long-term disability.^1,2^ Clinically, HIE is classified into mild, moderate, and severe, with moderate and severe cases carrying markedly higher risks of death and long-term neurodevelopmental sequelae.

Traditionally, infants with mild HIE were thought to have favorable outcomes. However, evidence challenges this view, showing that subtle cognitive and behavioral difficulties may become apparent by 2 years of age and can evolve over time.^3,4,5,6,7^ By school age, children with a history of mild HIE show measurable cognitive, behavioral, and motor difficulties, with outcomes that fall between those of healthy peers and children with moderate encephalopathy.^4,5^

Although therapeutic hypothermia has proven efficacy in moderate to severe HIE,^8^ strategies to improve outcomes in mild HIE remain undefined. A major challenge lies in the diagnosis of mild HIE within a few hours of birth, as the clinical spectrum and signs of neurological dysfunction vary substantially.^9^ At the milder end, the signs are subtle and transient and could be easily missed, whereas at the more severe end of mild, HIE may be misclassified as moderate or even severe HIE, leading to undertreatment or overtreatment.^9^ Elucidation of the molecular underpinnings of mild HIE could enable identification of diagnostic biomarkers for inclusion in point-of-care tests with the potential to improve early HIE diagnosis and stratification, thus paving the way for targeted neuroprotective interventions.

Gene expression provides a snapshot of the biological response to a specific disease, thus providing insight into the underlying disease mechanisms. Differentially expressed genes can be developed into a clinically useful point-of-care test.^10,11,12,13,14^ Whole-blood transcriptomics has identified biologically meaningful pathways in moderate or severe HIE,^15,16,17^ supporting its use for severity stratification.

In this study, we compared whole-blood gene expression at birth between infants with different severities of HIE and healthy control infants. Our aim was to identify early whole-blood transcriptomic signatures associated with mild HIE and to characterize the biological functions of differentially expressed genes. In addition, we examined temporal changes in gene expression over the first 72 hours after birth in infants with HIE compared with matched healthy control infants.

## Methods

### Study Design

This case-control study was approved by the Imperial College of London and University of Campania “Luigi Vanvitelli” ethics committees. The Strengthening the Reporting of Observational Studies in Epidemiology (STROBE) reporting guideline was followed.

We obtained written informed parental consent to collect blood samples for gene expression studies from infants recruited for 2 prospective studies. The mild HIE cohort was recruited in the early randomization arm of the COMET (Cooling in Mild Encephalopathy) pilot randomized clinical trial (n = 48; 14 cooled and 34 normothermia) in the UK and Italy between October 2019 and April 2023.^18^ The moderate-severe HIE cohort included 35 consecutive infants with moderate or severe HIE who underwent whole-body hypothermia and 39 matched healthy control babies, recruited at the University Hospital of Campania in Italy between January 2017 and June 2019^16^ and between September 2019 and 2025. The healthy control samples were sequenced in 2 batches: the first 14 infants alongside the moderate-severe HIE cohort^16^ and the remaining 25 infants alongside the mild HIE cohort (eTable 1 and eFigure 1 in Supplement 1).

### Clinical Assessments

The infants who were enrolled underwent a standardized neurological examination by one of the trained and certified assessors (S.T., P.M., and R.G.) using expanded modified Sarnat staging^19,20^ between 1 and 6 hours after birth.^21^ All recruited infants also underwent magnetic resonance imaging (MRI) using standardized protocols across sites between 4 and 14 days of age. A neurodevelopmental examination was performed by one of the certified examiners (S.T., P.M., and R.G.) for infants between 18 and 24 months of age using the Bayley Scales of Infant Development III and IV (eMethods in Supplement 1). MRI and neurodevelopmental outcome assessments were not performed for healthy control infants.

### Blood Collection and Analysis

Approximately 0.5 mL of whole blood was collected using customized RNA tubes (PAXgene; PreAnalytiX)^15,16^ within 6 hours after birth and before initiating therapeutic hypothermia and again at 72 hours. For infants receiving cooling, this later sample was collected during ongoing therapeutic hypothermia, whereas samples were collected from control patients and infants with mild HIE in the noncooling arm at normal body temperature (36.5-37.5 °C). Next-generation RNA sequencing was performed using 2 platforms: HiSeq 4000 (Illumina) for the infants with moderate or severe HIE alongside healthy control patients and NextSeq 2000 (Illumina) for the infants with mild HIE and their corresponding healthy control patients.

### Statistical Analysis

Differential expression analysis was conducted in R, version 4.5.0 (R Project for Statistical Computing). Differential expression was assessed by fitting linear models to each transcript and computing moderated *t* statistics. RNA sequencing data were analyzed as raw integer counts, consistent with the negative binomial modeling framework of DESeq2 package, version 1.50.2 (Bioconductor).

Infants with mild and moderate or severe HIE were compared with control patients and with each other, first in unadjusted models and then in models adjusted for birth weight, sex, and gestational age. Because all infants with moderate or severe HIE received therapeutic hypothermia, and infants with mild HIE received either hypothermia or usual care depending on randomization, the 72-hour comparison additionally adjusted for treatment arm. All statistical tests were 2 sided. Multiple testing was controlled using the Benjamini-Hochberg false discovery rate (FDR), with statistical significance defined as an FDR of less than 0.05. Data were analyzed from October 2025 to February 2026.

To evaluate temporal changes in gene expression, we fitted an interaction model including severity stage, time point (T0 vs T1; T0 corresponds to samples collected within 6 hours after birth and prior to initiation of therapeutic hypothermia, whereas T1 corresponds to samples obtained at 72 hours [day 3]), and their interaction, with adjustment for sex, birth weight, gestational age, and treatment arm. The stage × time point term tested whether expression trajectories over the first 72 hours differed between severity groups relative to control patients.

Pathway analysis was performed using the differentially expressed genes (DEGs; FDR <0.05), mapping them to biological functions within the Ingenuity Pathways Knowledge Base (Ingenuity Systems).^22^ To identify broader transcriptional shifts, global differential expression was evaluated using 1-way analysis of variance on batch-corrected log_2_ counts per million values across HIE severity groups, applying Bonferroni correction (*P* < .01) to derive a high-confidence minimal gene set for classification. Details of the sequencing method, quality control for batch effects, and analysis are provided in the eMethods and eFigures 2, 3, and 4 in Supplement 1.

## Results

The study included 112 full-term infants, including 73 infants with HIE (40 with mild HIE, 29 with moderate HIE, and 4 with severe) and 39 healthy control patients, with comparable baseline characteristics across groups. The median birth weight was 3.3 kg (IQR, 3.0-3.6 kg) in the moderate-severe HIE group, 3.3 kg (IQR, 3.1-3.5 kg) in the mild HIE group, and 3.4 kg (IQR, 3.0-3.6 kg) in control group. The median gestational age was 40.0 weeks (IQR, 39.0-40.6 weeks) in the moderate-severe group, 39.3 weeks (IQR, 39.0-41.0 week) in the mild group, and 39.4 weeks (IQR, 39.0-40.4 weeks) in the control group. The sex distribution was similar (female infants: 20 [61.0%] with moderate or severe HIE, 19 [47.5%] with mild HIE, and 23 [59.0%] control patients).

A total of 88 blood samples were collected shortly after birth (mean [SD] age of infants, 1.8 [1.5] hours) and 75 samples at 72 to 76 hours (Table; eTables 1 and 2 in Supplement 1). Two infants with moderate or severe HIE from the original cohort^16^ were excluded: 1 due to sampling outside predefined time windows and 1 due to failure of the transcriptomic data to pass preprocessing and harmonization. Of the 48 infants with mild HIE originally recruited, 8 (16.7%) were excluded because of inadequate RNA quality, leaving 40 infants in the final mild HIE dataset.

**Table.** Clinical Characteristics of Infants With HIE and Healthy Controls

Characteristic	Infants, No. (%)		
	Moderate or severe HIE (n = 33)	Mild HIE (n = 40)	Healthy controls (n = 39)
Birth and resuscitation			
Emergency cesarean delivery	15 (45.5)	11 (27.5)	0
Sex			
Female	20 (61)	19 (47.5)	23 (59.0)
Male	13 (39)	21 (52.5)	16 (41.0)
Birth weight, median (IQR), kg	3.3 (3.0-3.6)	3.3 (3.1-3.5)	3.4 (3.0-3.6)
Gestational age, median (IQR), wk	40.0 (39.0-40.6)	39.3 (39.0-41.0)	39.4 (39.0-40.4)
Umbilical cord pH, median (IQR)	6.9 (6.9-7.0)	7.0 (6.9-7.1)	NA
5-min Apgar score, median (IQR)	6 (5-8)	7 (6-8)	10 (9-10)
Intubation at birth	12 (36.4)	7 (17.5)	0
Neonatal hospitalization			
Induced hypothermia	33 (100)	12 (30.0)	NA
Seizures^a^	10 (30.3)	2 (5.0)	NA
Early-onset culture-positive sepsis	1 (3.0)	2 (5.0)	0
Invasive ventilation	22 (66.6)	7 (17.5)	0
Inotropic support	20 (60.6)	3 (7.5)	0
MRI			
MRI performed	31 (93.9)	38 (95.0)	0
Basal ganglia or thalamic injury detected	8 (24.2)	1 (2.5)	NA
White matter injury detected	10 (30.3)	10 (25.0)	NA
Cortical injury detected	4 (12.1)	1 (2.5)	NA
Outcomes at 18-22 mo			NA
Death	4 (12.1)	1 (2.5)	NA
Mild disability	0	3 (7.5)	NA
Moderate or severe disability	7 (21.2)	0	NA

Abbreviations: HIE, hypoxic-ischemic encephalopathy; MRI, magnetic resonance imaging; NA, not applicable.

^a^ Seizures 6 hours after birth.

All healthy control patients and infants with mild HIE in the noncooling arm maintained normal body temperature (36.5-37.5 °C). All infants with moderate or severe HIE received therapeutic hypothermia as standard care, and 12 of 40 infants with mild HIE (30.0%) were randomized to therapeutic hypothermia in the COMET trial.

### Clinical Course and Neurodevelopmental Outcomes

As expected, infants with moderate or severe HIE (n = 33) required more intensive support than those with mild HIE (n = 40). Invasive ventilation was needed for 22 of 33 infants with moderate or severe HIE (66.6%) vs 7 of 40 infants with mild HIE (17.5%) (difference, 49% [95% CI, 29.7%-69.3%]), and inotropic support was provided to 20 of 33 infants with moderate or severe HIE (60.6%) vs 3 of 40 infants with mild HIE (7.5%) (difference, 53.1% [95% CI, 34.9%-71.7%]). On MRI scans, basal ganglia or thalamic injury was detected 8 of 33 infants with moderate or severe HIE (24.2%) vs 1 of 40 infants with mild HIE (2.5%) (difference, 21.7% [95% CI, 6.5%-37.1%), white matter injury was detected in 10 of 33 infants with moderate or severe HIE (30.3%) vs 10 of 40 infants with mild HIE (25.0%) (difference, 5.3% [95% CI, −15.3% to 25.9%), and cortical injury was deteced in 4 of 33 infants with moderate or severe HIE (12.1%) vs 1 of 40 infants with mild HIE (2.5%) (difference, 9.6% [95% CI, −2.4% to 21.8%).

Death or moderate to severe disability at 18 to 22 months occurred for 11 of 33 infants (33.3%) in the moderate-severe HIE group compared with 1 of 40 infants (2.5%) in the mild HIE group. All control infants remained in the postnatal ward and did not have any complications during neonatal hospitalization. Further details are provided in the Table and eTables 1 and 2 in Supplement 1.

### Gene Expression Profile at Birth

**Figure 1.**
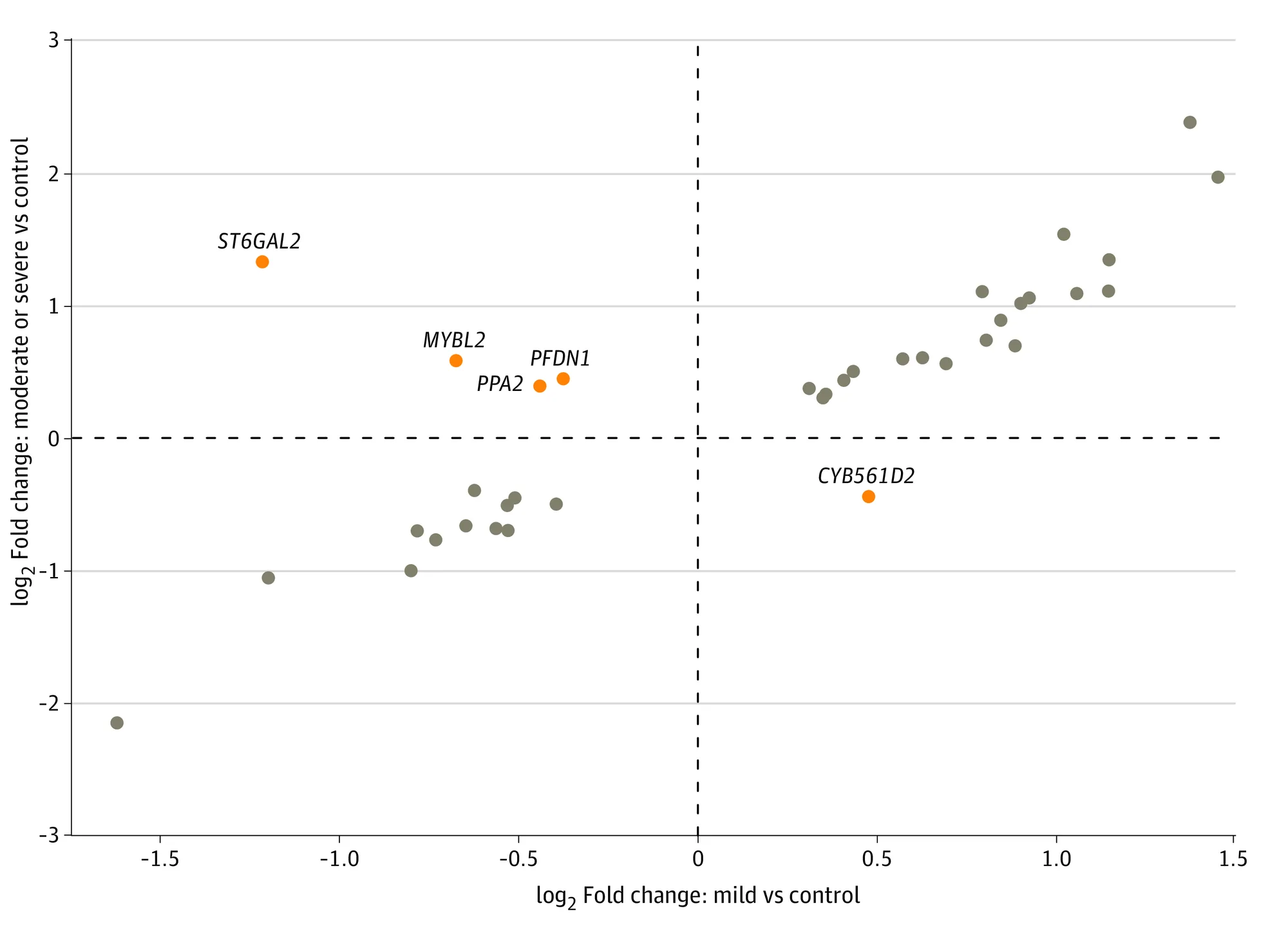
Scatterplot Showing Gene Expression Shifts Across Severity Levels of Hypoxic-Ischemic Encephalopathy (HIE) Scatterplot of log_2_ fold changes for the 37 shared genes, comparing infants with mild HIE (x-axis) and infants with moderate or severe HIE vs healthy control infants (y-axis). Only 5 genes exhibited opposing fold-change directions between the 2 severity groups.

The analysis included 88 infants with high-quality RNA samples, collected at a mean (SD) age of 1.8 (1.5) hours after birth, before the initiation of hypothermia when applicable. A total of 622 genes in the comparison of infants with moderate or severe HIE vs control infants and a total of 981 genes in the comparison of infants with mild HIE vs control infants were differentially expressed (FDR <0.05). Only 37 genes overlapped between these 2 comparisons, and 5 of these exhibited opposite directions of fold change in mild vs moderate or severe HIE (Figure 1). Direct comparison of mild vs moderate or severe HIE identified 3177 DEGs (FDR <0.05).

**Figure 2.**
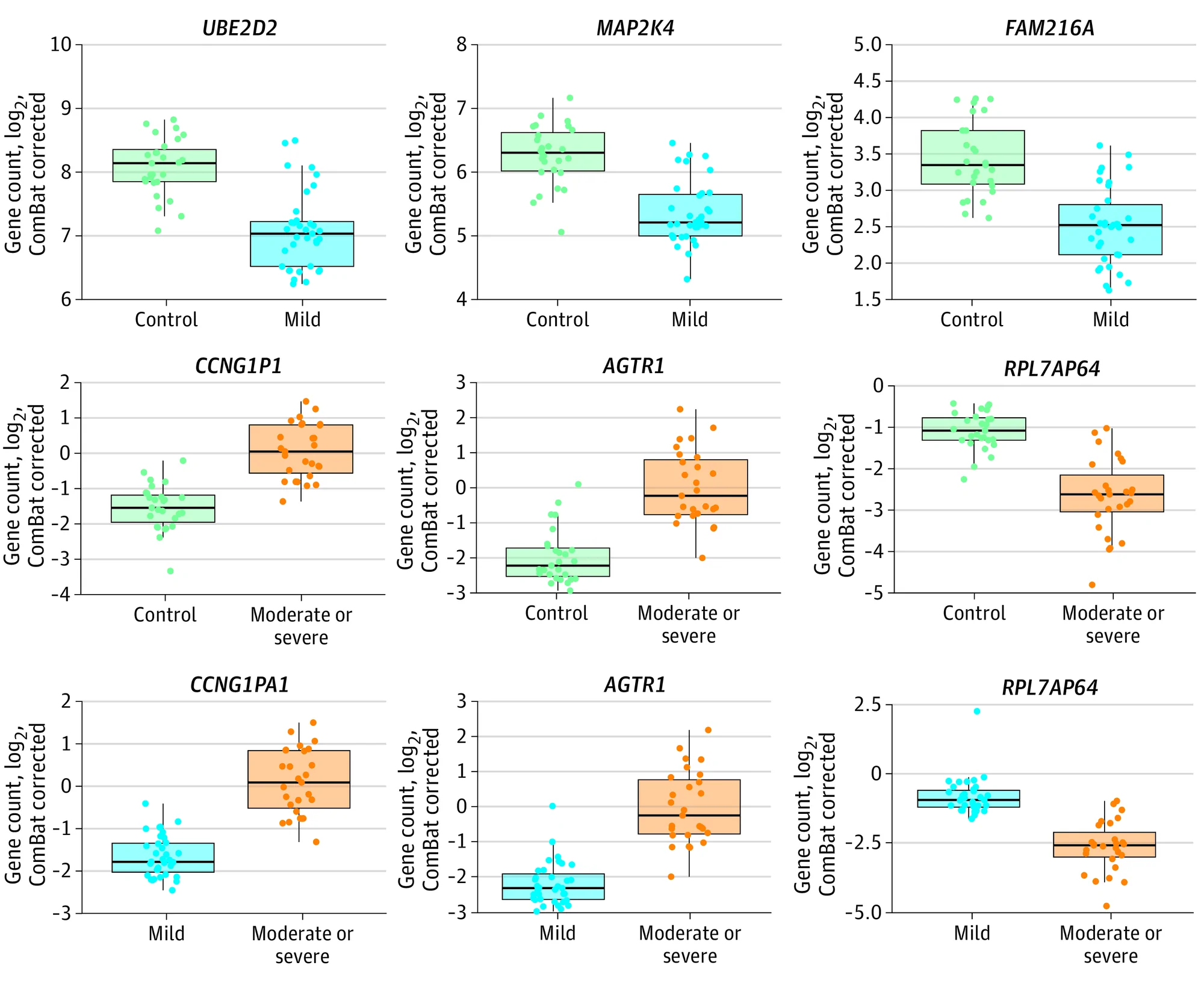
Box Plots Showing Expression of the Top 3 Differentially Expressed Genes for Each Pairwise Comparison Soon After Birth Box plots show log_2_-transformed, ComBat-corrected expression values for the top 3 genes in the comparison of infants with mild hypoxic-ischemic encephalopathy (HIE) vs control infants (top panel), of infants with moderate or severe HIE vs control infants (middle panel), and of infants with mild HIE vs infants with moderate or severe HIE (bottom panel). Each panel displays the distribution of gene expression across the 2 groups, with individual samples overlaid as points. Boxes represent the IQR, the horizontal line indicates the median, and whiskers extend to 1.5 × IQR. Differential expression statistics for the top genes in each comparison are presented. For infants with mild HIE vs control infants, the log_2_ fold change is −1.04 for *UBE2D2*, −0.93 for *MAP2K4* and *FAM216A* (all false discovery rates [FDRs] <0.001). For infants with moderate or severe HIE vs control infants, the log_2_ fold change is 1.61 for *CCNG1P1*, 1.95 for *AGTR1*, and −1.57 for *RPL7AP64* (all FDRs <0.001). For infants with mild HIE vs infants with moderate or severe HIE, the log_2_ fold change is −1.81 for *CCNG1P1*, −2.22 for *AGTR1*, and 1.79 for *RPL7AP64* (all FDRs <0.001).

The top DEGs in the comparison of infants with mild HIE vs control infants were *UBE2D2*, *MAP2K4*, and *FAM216A*. In the comparison of infants with moderate or severe HIE vs control infants, the top DEGs were *CCNG1P1*, *AGTR1*, and *RPL7AP64*. In the comparison of infants with mild HIE vs those with moderate or severe, the top DEGs were *CCNG1P1*, *AGTR1*, and *RPL7AP64* (Figure 2; eTables 3, 4, and 5 in Supplement 2).

Pathway enrichment analysis was performed using all differentially expressed genes and stratified into pathways unique to moderate or severe HIE, unique to mild HIE, or shared between both groups (eTable 6 in Supplement 2). Moderate or severe HIE showed a striking and highly specific enrichment of adaptive and innate immune activation pathways. The strongest enrichment was observed for autoimmune thyroid disease signaling (*z* score not determined; *P* < .001), indicating significant overrepresentation of pathway genes among the differentially expressed genes compared with the expressed gene background. No regulation *z* score was determined because there was no sufficient directional information for activation prediction. In contrast, pathways unique to mild HIE were dominated by cell-intrinsic stress responses, including cell-cycle regulation, DNA repair, mitochondrial and metabolic pathways, proteasome or ubiquitin signaling, and RNA processing. The top pathway unique to mild HIE was the processing of capped intron-containing pre-mRNA (*z* score, −0.68; *P* < .001). Pathways shared between mild HIE and moderate or severe HIE included broad innate immune responses, antigen presentation, and core metabolic pathways.

**Figure 3.**
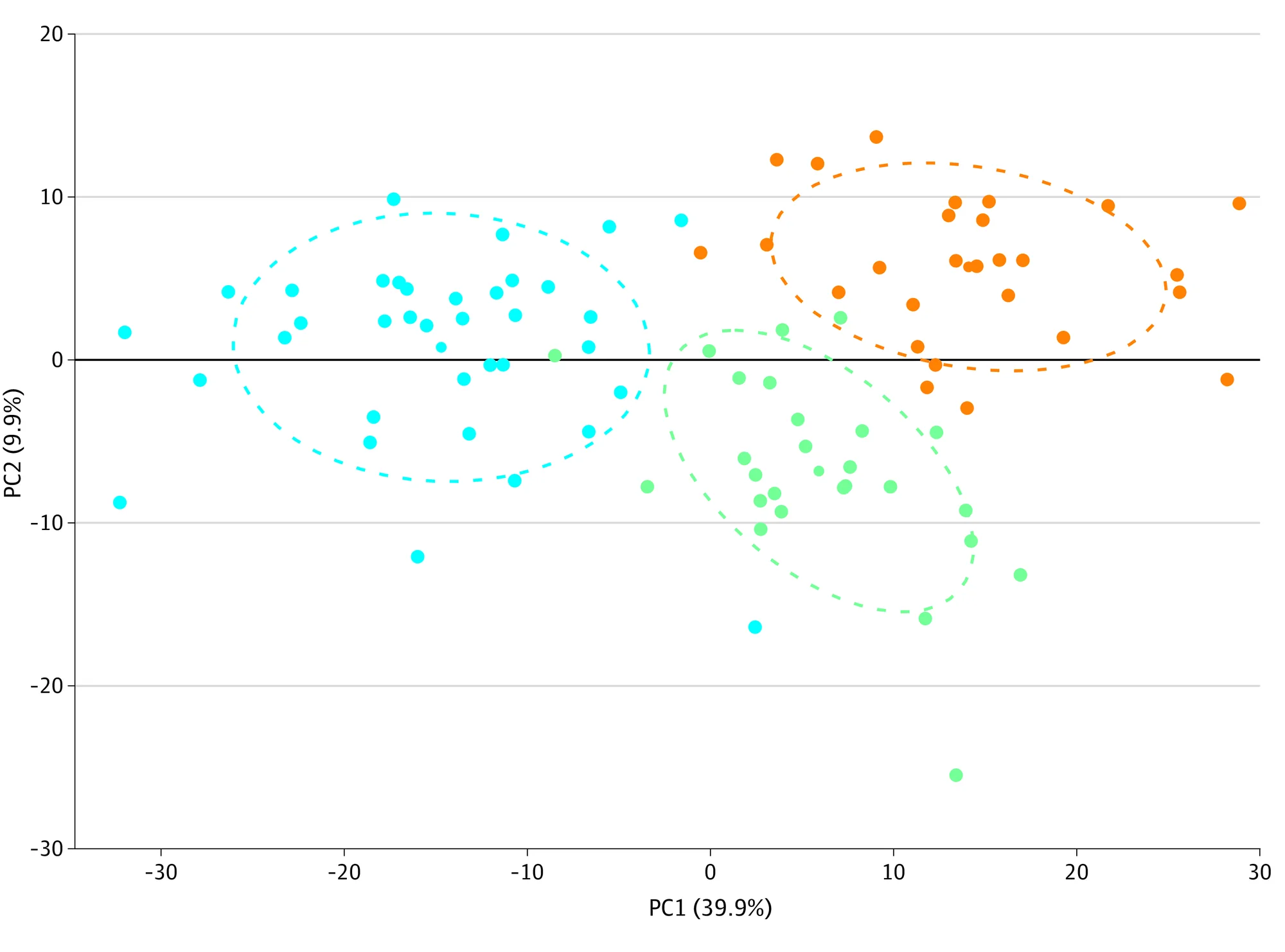
Scatterplots Showing Principal Component Analysis of Severity-Associated Gene Expression Profiles Principal component analysis of whole-transcriptome profiles based on the 526 genes identified as significant in a 3-way analysis of variance comparison across infants with mild hypoxic-ischemic encephalopathy (HIE) (blue), infants with moderate or severe (orange) HIE, and healthy control infants (green) (Bonferroni-adjusted *P* < .01). The first 2 principal components (PC1 and PC2) show clear separation of the 3 clinical groups, indicating that these genes capture the major variance associated with HIE severity.

When directly comparing infants with mild vs infants with moderate or severe HIE, the most enriched pathway was the cell-cycle checkpoints (*z* score, −4.50; *P* < .001). The list of all the significant pathways for each comparison is provided in eTables 7, 8, and 9 in Supplement 2. When a 3-way comparison was performed with analysis of variance, optimal separation occurred with the 526 significant genes (Bonferroni adjusted *P* < .01; Figure 3).

### Gene Expression Profile at 3 Days

The analysis included 74 infants from whom high-quality RNA was obtained from whole blood at a mean (SD) age of 74.3 (1.85) hours after birth (eTable 2 in Supplement 1). These later samples from cooled infants were collected during active hypothermia, whereas samples were collected at normothermia from control infants and infants with normothermic mild HIE. For infants with mild HIE who underwent therapeutic hypothermia, samples were likewise collected during active cooling.

**Figure 4.**
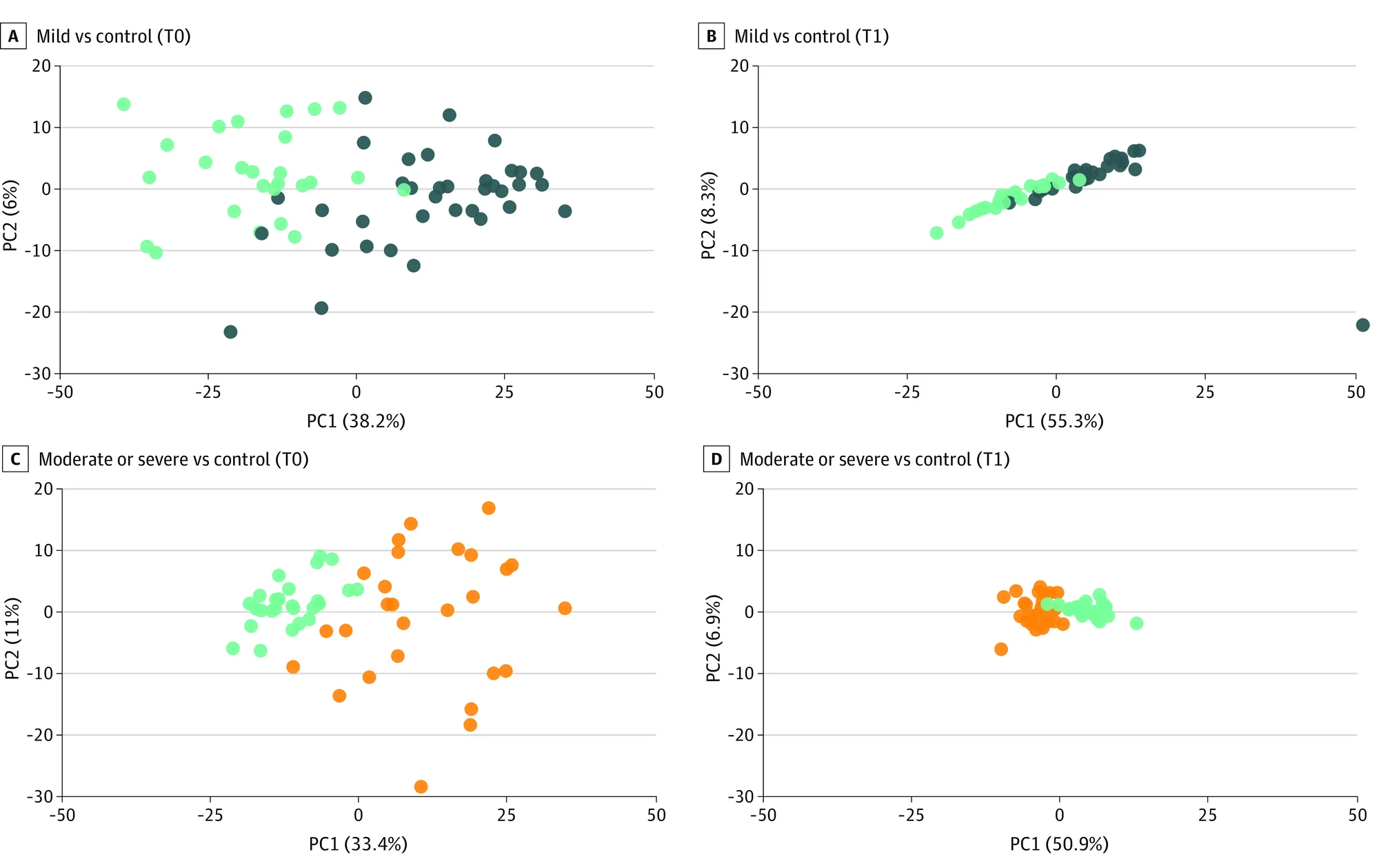
Scatterplots Showing Temporal and Severity-Dependent Gene Expression Changes in Neonatal Encephalopathy The first 2 principal components (PC1 and PC2) are presented in the principal component analysis of the differentially expressed genes among infants with mild hypoxic-ischemic encephalopathy (HIE) (blue), infants with moderate or severe HIE (orange), and healthy control infants (green) at 2 postnatal time points: immediately after birth (T0) and at 72 hours (T1).

A total of 54 genes in the comparison of infants with moderate or severe HIE vs control infants and a total of 242 genes in the comparison of infants with mild HIE vs control infants were differentially expressed (FDR <0.05). Direct comparison of mild vs moderate or severe HIE identified 386 DEGs (FDR <0.05) (eTables 10, 11, and 12 in Supplement 2). Compared with the extensive transcriptomic disturbance observed soon after birth, gene expression at 72 hours showed a smaller number of differentially expressed genes, and global expression profiles were substantially closer to those of control infants (Figure 4; eFigure 5 in Supplement 1).

The top DEGs in the comparison of infants with mild HIE vs control infants were *TRAPPC10*, *CDK2*, and *PTGES3P1*. In the comparison of infants with moderate or severe HIE vs healthy control infants, the top DEGs were *CALHM6*, *TNRC18P1*, and *IGKV3-20*. In the comparison of infants with mild HIE vs those with moderate or severe HIE, the top DEGs were *LGALS9C*, *TREML3P*, and *FANK1* (eFigure 6 in Supplement 1).

At 72 hours, moderate or severe HIE remained enriched for immune-activation pathways, whereas mild HIE continued to show enrichment of cell-intrinsic repair and metabolic resilience pathways. The top mild-specific pathway was microRNA biogenesis (*z* = −2.24; *P* < .001), consistent with ongoing checkpoint engagement and transcriptional stabilization. When directly comparing infants with mild HIE vs infants with moderate or severe HIE, the infants with moderate or severe HIE showed marked enrichment of immune-driven pathways (eTables 13, 14, 15, and 16 in Supplement 2).

### Temporal Changes in Gene Expression

We then studied how gene expression changes over time according to the severity stage of HIE, by comparing the gene expression in HIE against the age-matched healthy control infants (eTable 2 in Supplement 1). Thirty genes were differentially expressed over time in infants with moderate or severe HIE compared with control infants, indicating expression trajectories that differed from those of control infants. In contrast, only 1 gene showed a significant temporal change in infants with mild HIE. These findings suggest that, relative to control infants, infants with mild HIE exhibit minimal transcriptomic change over the first 72 hours, consistent with rapid biological normalization.

## Discussion

In this study, infants with mild HIE exhibited a distinct host gene expression profile compared with both infants with moderate or severe HIE and healthy control infants in peripheral blood samples obtained shortly after birth and before initiation of therapeutic hypothermia. Moreover, unlike infants with moderate or severe HIE, the gene expression profile of infants with mild HIE became similar to that of healthy control infants by day 3 of life, suggesting rapid biological normalization. To our knowledge, this study represents the first gene expression profiling of mild HIE in a well-characterized cohort.

Mild HIE was characterized by modest downregulation of stress-responsive genes and relative preservation of metabolic signaling, consistent with a more adaptive cellular state. In contrast, moderate and severe HIE showed marked upregulation of injury-associated genes, including *AGTR1* and *CCNG1P1*, together with suppression of ribosomal and metabolic transcripts, indicating a shift toward inflammation, oxidative stress, and impaired cellular homeostasis.^23,24^ These data suggest that gene expression profiles mirror the clinical spectrum of encephalopathy and may enable rapid disease stratification based on underlying systemic biological mechanisms and severity.

Early and accurate diagnosis of HIE remains essential to ensure timely delivery of neuroprotective therapy. For more than 50 years, the classification of encephalopathy severity has been based on assessment of neurological status during the first 3 days after birth.^25^ However, this clinical classification is inherently subjective and can be influenced by concurrent treatments, such as sedation, or comorbidities, including infection. The definition of mild HIE has become critically important because of uncertainty regarding the safety and benefit of therapeutic hypothermia in this population, in contrast to infants with moderate or severe HIE, for whom hypothermia is the standard of care.^9,18^ Our data demonstrate that gene expression profiling can distinguish infants with mild HIE from those with moderate or severe HIE and from healthy control infants shortly after birth, and that these distinctions reflect meaningful underlying biological differences. However, determining whether these patterns can reliably distinguish clinical groups will require validation in larger cohorts.

Pathway analysis reinforced the divergence in biological responses between mild and moderate or severe HIE. Mild HIE was characterized by enrichment of cell-intrinsic adaptive programs, including RNA processing, cell-cycle regulation, DNA-repair pathways, and metabolic regulation highlighting a transcriptional response centered on maintaining RNA maturation and genomic stability, consistent with preserved cellular homeostasis. Additional mild-unique pathways included mitochondrial and metabolic pathways, collectively suggesting adaptive resistance to short-term hypoxic-ischemic stress through stabilization of transcriptional, metabolic, and proteostatic networks.^26,27,28^ In contrast, moderate or severe HIE demonstrated a highly specific and coordinated immune-activation signature, with exclusive enrichment of T-cell receptor signaling, antigen-presentation pathways, cytokine and chemokine signaling, and apoptosis-associated modules. This pattern suggests that moderate or severe HIE is accompanied by systemic immune activation and proteotoxic stress, with disruption of intracellular homeostasis. Together, these findings suggest that, at the systemic level, mild HIE is associated with relative preservation of transcriptional and metabolic homeostasis, whereas moderate or severe HIE is accompanied by a more pronounced inflammatory and proteotoxic stress response. Given that these data are derived from whole blood, they should be interpreted as reflecting systemic responses to hypoxia-ischemia and multiorgan injury, rather than as a direct readout of cerebral molecular pathology.

Direct comparison of mild HIE vs moderate or severe HIE further emphasized these mechanistic differences. Hypoxia normally triggers a shift toward glycolysis and suppression of energy-intensive processes.^29^ In mild HIE, preserved cell-cycle and mitochondrial pathways indicate maintained metabolic competence and coordinated adaptive repair. In contrast, moderate or severe HIE shows suppression of these cell-intrinsic programs with marked activation of inflammatory and proteotoxic-stress signaling, consistent with metabolic failure and loss of transcriptional homeostasis. This pattern reinforces the view that mild HIE maintains a reparative, energy-competent state, whereas moderate or severe HIE reflects a transition to systemic inflammatory activation and impaired cellular resilience.

Brain injury after HIE evolves over the first few days after birth through primary, secondary, and tertiary phases, although the evolution in mild HIE is less well characterized.^30^ Rapid quantification of early injury dynamics may help monitor therapeutic response and identify targets for intervention. Previous evidence has shown that with increasing severity of outcomes, there is progressive upregulation of hypoxia-related and oxidative-stress–related pathways after moderate and severe HIE.^16^ The present data align with these observations and show persistent immune dysregulation and calcium signaling disruption (*CALHM6* and *IGKV3-20*) in moderate and severe HIE. In contrast, only a single gene in mild HIE showed a significant interaction between HIE severity and time, indicating that gene expression trajectories over the first 72 hours were largely similar to those of control infants. Consistent with this, the cross-sectional analysis at 72 hours showed a near-complete convergence of mild HIE expression profiles with those of healthy control infants. Together, these findings suggest rapid biological normalization in mild HIE and highlight the dynamic, severity-dependent nature of the neonatal transcriptomic response to hypoxia-ischemia. Although transcriptomic profiles in mild HIE normalized by 72 hours, transient early molecular disturbances may still carry developmental relevance. Even short-lived alterations in stress-response, metabolic, or synaptic pathways can trigger downstream changes that persist beyond the period in which peripheral gene expression appears normal. Thus, rapid transcriptomic normalization does not exclude later neurodevelopmental variability and supports the need for continued follow-up for infants with mild HIE.

Complementary metabolomic and epigenetic studies demonstrated severity-dependent alterations in mitochondrial energetics, redox balance, and hypoxia-responsive regulation, aligning closely with the transcriptomic patterns observed here and supporting a convergent multiomic model of HIE biology.^31,32,33,34,35^ Their integration may therefore provide an additive and biologically grounded framework for early stratification of HIE severity and for identifying infants most likely to benefit from targeted neuroprotective interventions.

### Limitations

Although sampling procedures were standardized to minimize confounding, several limitations should be noted. First, gene expression was measured in whole blood and therefore reflects systemic rather than brain-specific responses; peripheral signatures provide insight into injury biology but cannot fully capture cerebral processes. Second, although we adjusted for temperature status at 72 hours, residual confounding from clinical care pathways cannot be excluded. Third, more DEGs were identified in mild HIE than in moderate or severe HIE, likely reflecting both biology and statistical power; circulating cells in mild HIE retain metabolic reserve to mount coordinated transcriptional responses, whereas moderate or severe HIE is characterized by energy failure and transcriptional suppression. The mild HIE group was also larger and more homogeneous, whereas the moderate or severe HIE group was smaller and included few infants with severe HIE; thus, our analyses were not powered to distinguish moderate from severe HIE, and the combined group should be viewed as a spectrum of more advanced encephalopathy. Although sequencing was performed on 2 platforms, control infants were analyzed jointly, and the distinct pathway signatures argue against platform artifact. The functional significance of some DEGs, particularly pseudogenes and less-characterized transcripts, remains uncertain, and larger multiomics studies will be required to validate these findings.

## Conclusions

In this case-control study, distinct transcriptomic signatures within hours of birth indicated rapid gene expression changes even when neurological signs were subtle. Molecular profiling may complement the modified Sarnat examination by providing an objective measure of systemic responses to hypoxia-ischemia, particularly in mild HIE where presentation is heterogeneous. The rapid normalization of mild HIE profiles, contrasted with persistent dysregulation in moderate or severe HIE cases, reflects the biological distinction between transient mild encephalopathy and sustained metabolic and inflammatory stress. Blood-based transcriptomic markers may support personalized care by identifying infants whose biological trajectory diverges from their initial clinical presentation. Integration of transcriptomic, metabolomic, and epigenetic markers may further enhance early risk stratification and guide timely neuroprotective strategies.

## Notes

Supplement 1.
eMethods.
**eTable 1**. Clinical Variables of HIE Infants in Batches 1 and 2 **eTable 2**. Mean (SD) Age and Mean (SD) Body Temperature at Blood Sample Collection **eFigure 1**. Flow Diagram of the Study **eFigure 2**. Batch Effect Correction **eFigure 3**. Identification of Any Sources of Variance **eFigure 4**. Expression of the Top Three Differentially Expressed Genes for Each Pairwise Comparison at 72 Hours

Supplement 2. **eTable 3.** Differentially Expressed Genes Associated with Mild HIE (Comparison Mild HIE versus Healthy Controls, FDR < 0.05) **eTable 4.** Differentially Expressed Genes Associated with Moderate or Severe HIE (Comparison Moderate or Severe HIE versus Healthy Controls, FDR < 0.05) **eTable 5.** Differentially Expressed Genes in The Comparison Mild versus Moderate or Severe HIE (FDR < 0.05) **eTable 6.** Ingenuity Pathway Analysis Results Showing Canonical Pathways Classified as Unique to Moderate/Severe HIE, Unique to Mild HIE, or Shared Between Both Groups at T0 (Adjusted) **eTable 7.** Full Ingenuity Pathway Analysis (IPA) Results for All Canonical Pathways Enriched in Mild HIE vs Controls at T0 (Adjusted) **eTable 8.** Full Ingenuity Pathway Analysis Results for All Canonical Pathways Enriched in Moderate/Severe HIE vs Controls at T0 (Adjusted) **eTable 9.** Full Ingenuity Pathway Analysis Results for All Canonical Pathways Enriched in Mild vs Moderate/Severe HIE at T0 (Adjusted) **eTable 10.** Differentially Expressed Genes Associated with Mild HIE at 72 Hours (Comparison Mild HIE versus Healthy Controls, FDR < 0.05) **eTable 11.** Differentially Expressed Genes Associated with Moderate or Severe HIE at 72 Hours (Comparison Moderate or Severe HIE versus Healthy Controls, FDR < 0.05) **eTable 12.** Differentially Expressed Genes in the Comparison Mild versus Moderate or Severe HIE at 72 Hours (FDR < 0.05) **eTable 13.** Ingenuity Pathway Analysis Results Showing Canonical Pathways Classified as Unique to Moderate/Severe HIE, Unique to Mild HIE, or Shared Between Both Groups at T1 (adjusted) **eTable 14.** Full Ingenuity Pathway Analysis (IPA) Results for All Canonical Pathways Enriched in Mild HIE vs Controls at 72 Hours (Adjusted) **eTable 15.** Full Ingenuity Pathway Analysis Results for All Canonical Pathways Enriched in Moderate/Severe HIE vs Controls at 72 Hours (Adjusted) **eTable 16.** Full Ingenuity Pathway Analysis Results for All Canonical Pathways Enriched in Mild Vs Moderate/Severe HIE at 72 Hours (Adjusted)

Supplement 3.
Data Sharing Statement
