# Supplementary material for: Whole-Blood Gene Expression and Hypoxic-Ischemic Encephalopathy at Birth
Source: JAMA Netw Open. 2026 Aug 21;9(8):e2629378. doi: 10.1001/jamanetworkopen.2026.29378 (PMC13499004; doi:10.1001/jamanetworkopen.2026.29378)
Supplement: Supplementary file 1 [file jamanetwopen-e2629378-s001.pdf]

Supplemental Online Content

Montaldo P, Herberg J, Uppal S, et al. Whole-blood gene expression and hypoxic-ischemic encephalopathy at birth. *JAMA Netw Open*. 2026;9(8):e2629378. doi:10.1001/jamanetworkopen.2026.29378

eMethods.

eTable 1. Clinical Variables of HIE Infants in Batches 1 and 2

eTable 2. Mean (SD) Age and Mean (SD) Body Temperature at Blood Sample Collection

eFigure 1. Flow Diagram of the Study

eFigure 2. Batch Effect Correction

eFigure 3. Identification of Any Sources of Variance

eFigure 4. Expression of the Top Three Differentially Expressed Genes for Each Pairwise Comparison at 72 Hours

This supplemental material has been provided by the authors to give readers additional information about their work.

© 2026 Montaldo P et al. *JAMA Network Open.*

eMethods.

Healthy controls

Neonates who required blood group typing and serum bilirubin checks, as per the local policy, were recruited as healthy controls. Neonates with risk factors for sepsis or requiring antibiotic therapy were not eligible.

Outcome definition

Severe disability was defined as any of the following: a Bayley III cognitive score < 70; a Gross Motor Function Classification System (GMFCS) level of 3 to 5; blindness; or hearing loss (inability to understand commands despite Amplification). Moderate disability was defined as a Bayley III cognitive score of 70 to 84 and either a GMFCS level of 2, seizure disorder or a hearing deficit requiring amplification to understand commands.

RNA sample extraction, processing and quality assessment

Peripheral venous or arterial blood (0.5 mL) was added to 1.4 mL ribonucleic acid -stabilizing solution (PreAnalytiX, Qiagen/BD) as per the manufacturer's instructions. The samples were initially stored at -20 °C and then transferred to the central laboratory at -80° C. The RNA extraction procedure for all the samples was performed centrally, and each RNA sample was quantified using Qubit and run on a Tapestation to assess sample integrity. High-quality RNA was defined as having an RNA integrity number (RIN) ≥ 7, and only samples meeting this threshold were included in downstream analyses. Ribosomal and mitochondrial RNA were removed via ribodepletion. After fragmentation, the sample was converted to double stranded DNA and ligated to Illumina adapters. Tapestation was used to assess fragment distribution and presence of artefacts.

The blood samples were sequenced in two different batches. The first batch included 33 infants with moderate or severe hypoxic ischaemic encephalopathy and 14 healthy controls[14]. The RNA samples from batch 1 were sequenced (Next generation sequencing) on an Illumina HiSeq4000 to generate 50M reads/sample (Imperial BRC Genomics Facility, Imperial College London, UK). The second batch included 40 infants with mild HIE and 25 healthy controls. The RNA samples from batch 2 were sequenced on a NextSeq 2000 to generate 30M reads/sample (Instituto De Investigacion Sanitaria La Fe, Spain). The RNA from 1 healthy control and 1 infant with mild HIE were sequenced twice for internal quality check and consistency.

Forward and reverse reads were quality checked using the program fastp (v0.23.4) applying a tail cut searching for a minimum of Q30 in a window of 20 nucleotides. Reads shorter than 50 bp were removed from the analysis. Reference genome (Homo sapiens, GRCh38) was indexed and mapped using the program STAR (v2.7.11b). Feature counts represent the number of sequencing reads that align to each genomic feature (typically exons or genes). These were generated using htseq-count (v2.0.8), which assigns each aligned read to a gene based on its overlap with annotated features in the reference genome, producing integer gene-level counts for downstream differential expression analysis. Reads are assigned according to the gene_id field in the Gene Transfer Format (GTF) annotation, with

© 2026 Montaldo P et al. *JAMA Network Open.*

counts summed across all transcripts belonging to the same gene. In practice, this collapses transcript-level reads into a single gene-level count for each gene.

Analyses

All analyses were performed using R (version 4.5.0). Principal component analysis (PCA) was used as part of the initial quality control to assess sample clustering and detect potential sources of technical variation.

Raw gene-level integer count matrices from all sequencing runs were merged into a single expression matrix, and individual-level metadata from two independent sources were harmonised prior to analysis. Genes with low expression were removed before downstream processing. Low expression was defined as fewer than 10 total counts across all samples, and genes below this threshold were excluded prior to downstream processing.

Library sizes were normalised using the trimmed mean of M values (TMM) method to account for compositional differences across samples. Normalised counts were then transformed to continuous $\log_2$ counts per million ($\log_2$-CPM) with associated precision weights using the *voom* function in limma (v3.60.6), which estimates the mean–variance relationship in RNA-seq data to generate weighted $\log_2$-CPM values suitable for linear modelling, based on a design matrix including hypoxic–ischaemic encephalopathy (HIE) severity stage, sex, birthweight, and gestational age. Gestational age and birthweight were modelled as continuous covariates. PCA plots coloured by these variables showed smooth monotonic gradients without curvature or multimodal structure, indicating no evidence of strong non-linear relationships (**eFigure 2**). Consistent with this, spline-based model comparisons (joint F-tests, AIC/BIC) did not support non-linear effects. Linear terms were therefore used in the final differential expression model. This approach models the mean–variance relationship in RNA-seq data and enables the use of linear models with empirical Bayes–moderated t-statistics. In this context, moderated t-statistics refer to t-tests in which each gene's variance estimate is stabilised by borrowing information across all genes. Instead of estimating variance for each gene independently—which can be noisy in modest sample sizes—the empirical Bayes procedure shrinks gene-specific variances toward a common value. This produces more stable and reliable test statistics, particularly for genes with low or variable counts.

To mitigate batch and platform effects while preserving biological signal, we applied ComBat from the sva package (v3.52.0) to the voom-transformed expression matrix, using sequencing batch as the adjustment variable and including HIE severity, birthweight, sex, and gestational age in the model matrix. The resulting continuous, batch-corrected $\log_2$-CPM expression values were used for all primary analyses. This approach removes batch-associated variation while protecting variation attributable to the biological factors of interest. PCA performed after ComBat correction confirmed effective removal of batch structure (**eFigure 3**), and additional PCA plots were used to visualise variance associated with gestational age, sex, and birthweight (**eFigure 2**). Differences in proportions between groups were summarised as absolute risk differences with 95% confidence intervals calculated using the standard method implemented in R prop.test() function, which applies a normal-approximation (Wald-type) interval to the risk difference.

Differential expression analysis was performed using linear modelling with empirical Bayes moderation in limma. PCA demonstrated that severe cases clustered within the moderate group, with no evidence of forming a distinct transcriptomic subgroup (**eFigure 4**). Therefore, infants with moderate and severe HIE were combined, and severity was collapsed into three levels (Control, Mild, Moderate/Severe) and used as the primary grouping factor for all analyses. Models were first fitted unadjusted and subsequently adjusted for gestational age, sex, and birthweight. For the analysis at 72 hours, hypothermia treatment (therapeutic hypothermia vs usual care) was additionally included as a covariate, as some infants with mild HIE were undergoing therapeutic hypothermia at the time of sampling. All samples were collected shortly after birth and before any therapy was initiated. Pre-specified contrasts included mild vs control, moderate/severe vs control, and mild vs moderate/severe.

To assess robustness to batch and platform effects, we performed a sensitivity analysis using an alternative modelling strategy. In the sensitivity analysis, we repeated the differential expression on voom-transformed, uncorrected data while including batch as an explicit covariate in the linear model. The alternative modelling strategy yielded results that were the same as the primary analysis, with log fold-changes showing near-perfect concordance between approaches (mild $r = 0.9996$; moderate/severe $r = 0.9998$) and complete overlap of all genes significant at FDR < 0.05 in the batch-covariate model (580/580 for mild; 980/980 for moderate/severe).

To assess whether temporal changes in gene expression differed by HIE severity, we fitted an interaction model including severity stage, timepoint (T0 vs T1), and their interaction, with adjustment for sex, birthweight, gestational age, and treatment arm. Infants were classified according to treatment allocation (therapeutic hypothermia vs usual care), and this binary temperature status at 72 hours was included as a covariate. The Stage × Timepoint interaction term tested whether expression trajectories over the first 72 hours diverged between severity groups relative to controls. Differentially expressed genes were defined as those with a false discovery rate (FDR)–adjusted P value < .05 for the contrast of interest.

In the hypothermia group, rectal and skin temperatures were monitored continuously throughout cooling, whereas in the normothermia group skin temperature was recorded hourly for the first 4 hours and every 2 hours thereafter.

Pathway enrichment analysis was performed using Ingenuity Pathway Analysis (IPA, QIAGEN), restricting input to genes meeting an FDR < 0.05. Canonical pathway significance was assessed using Fisher's exact test, and pathways were ranked by P value. IPA's regulation z-score algorithm was used to infer the predicted activation state (activation or inhibition) of each biological function. In this context, the regulation z-score reflects the predicted activation state of a pathway: positive values indicate activation, negative values indicate inhibition, and values close to zero indicate no consistent directional signal.

For each severity group, pathway enrichment was performed separately using the corresponding list of differentially expressed genes. Pathways were considered "unique" to mild or moderate/severe HIE if they were significantly enriched ($-\log_{10} P \geq 1.3$, equivalent to $P < .05$) in one group only, and "shared" if they met this threshold in both groups. Ingenuity Pathway Analysis (IPA) was selected because it provides curated, directionally annotated pathways and upstream regulator predictions, which are well suited to interpreting modest gene sets in clinical transcriptomic studies. Pathways were interpreted according to their

© 2026 Montaldo P et al. *JAMA Network Open.*

underlying biological functions rather than their disease-specific names, and all canonical pathways were retained to avoid introducing selection bias.

eTable 1. Clinical variables of HIE infants in batches 1 and 2.

	**Participants, No. (%)**			
	**Batch 1**		**Batch 2**	
**Characteristic**	**Moderate/ severe (n = 33)**	**Healthy controls (n = 14)**	**Healthy controls (n = 25)**	**Mild cohort (n = 40)**
**Neonatal**				
Emergency cesarean delivery	15 (45.5)	0	0	11 (27.5)
Sex				
Female	20 (61.0)	9 (64.3)	14 (56.0)	19 (47.5)
Male	13 (39.0)	5 (35.7)	11 (44.0)	21 (52.5)
Birth weight, median (IQR), kg	3.3 (3.0-3.6)	3.4 (3.2-3.7)	3.4 (2.9-3.5)	3.3 (3.1-3.5)
Gestational age, median (IQR), wk	40.0 (39.0-40.6)	39.2 (38.9-40.4)	39.4 (39-40.4)	39.3 (39-41)
Umbilical cord pH, median (IQR)	6.92 (6.9-7.0)	NA	NA	7.0 (6.9-7.1)
5-min Apgar score, median (IQR)	6 (5-8)	10 (9-10)	10 (10-10)	7 (6-8)
Intubation at birth	12(36.4)	0	0	7 (17.5)
**Clinical course**				
Induced hypothermia (sample collection within 6 h after birth)	33 (100)	NA	NA	12 (30.0)
Seizures[a]	10 (30.3)	NA	NA	2 (5.0)
Early-onset culture-positive sepsis	1 (3.0)	0	0	2 (5.0)
Need for invasive ventilation	22 (66.6)	0	0	7 (17.5)
Need for inotropic support	20 (60.6)	0	0	3 (7.5)
**MRI**				
MRI available	31 (93.9)	0	0	38 (95.0)
Basal ganglia or thalamic injury	8 (24.2)	NA	NA	1 (2.5)
White-matter injury	10 (30.3)	NA	NA	10 (25.0)
Cortical injury	4 (12.1)	NA	NA	1 (2.5)
**Outcomes at 18-22 mo**		NA	NA	
Death	4 (12.1)	NA	NA	1 (2.5)
Mild disability	0	NA	NA	3 (7.5)
Moderate or severe disability	7 (21.2)	NA	NA	0
Death and moderate or severe Disability	11 (33.3)	NA	NA	1 (2.5)

[a] Seizures after 6 hours after birth

© 2026 Montaldo P et al. *JAMA Network Open.*

eTable 2. Mean (SD) age at blood sample collection and number of samples.

	**T0[a], age (hours), mean (SD)**	**T1[b], age (hours), mean (SD)**	**Infants with 2 time points**	**Infants contributing only T0**	**Infants contributing only T1**	**Total infants sampled (T0 / T1)**
**Moderate/Severe HIE**	2.0 (1.4)	75.0 (2.4)	22	5	6	27 / 28
**Mild HIE**	1.3 (1.2)	76.0 (1.0)	20	16	4	36 / 24
**Healthy control neonates**	2.1 (1.7)	72.0 (2.1)	8	17	14	25 / 22

[a]T0 = Samples within six 6 hours after birth and before initiating therapeutic hypothermia.
[b]T1= Samples at 3 days. For infants receiving cooling, this later sample was collected during ongoing therapeutic hypothermia, whereas controls and infants with mild HIE in the non-cooling arm were sampled at normal body temperature (36.5-37.5 C°).

© 2026 Montaldo P et al. *JAMA Network Open.*

eFigure 1. Flow diagram of the study.

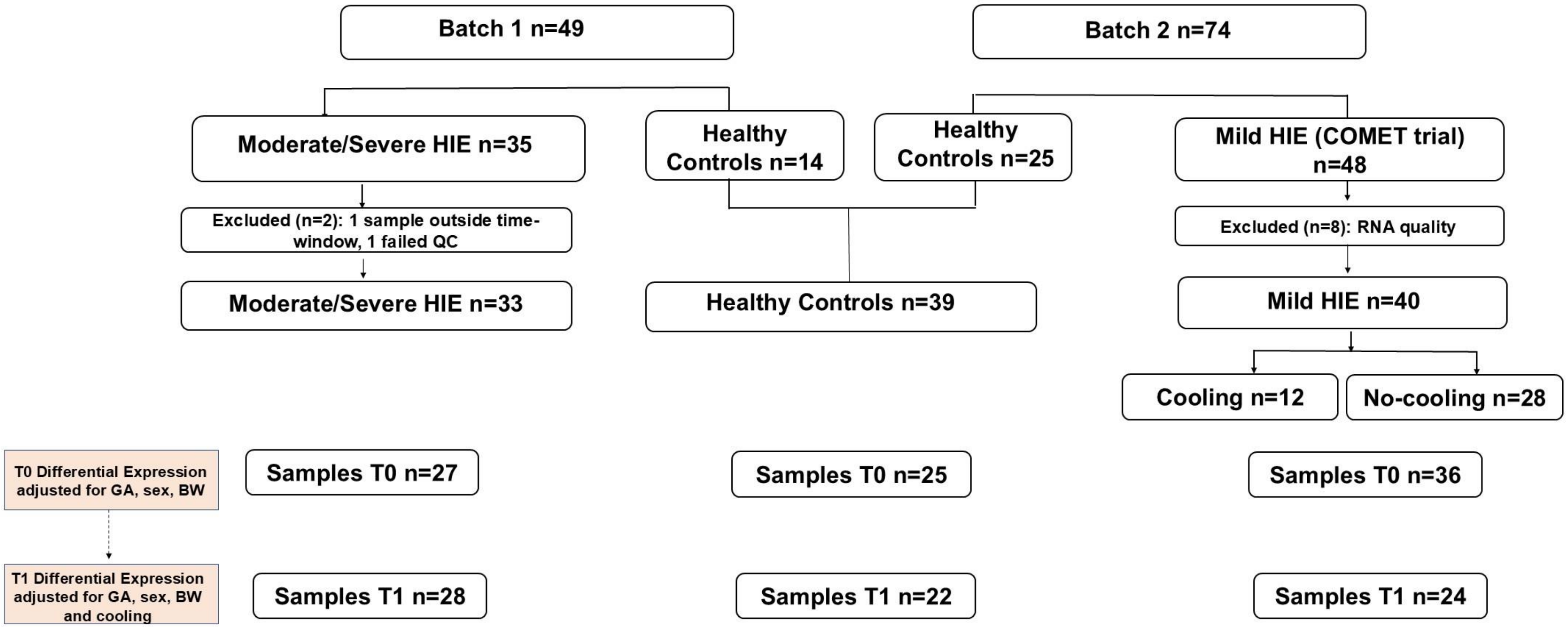

Definitions:**T0 =** Samples within 6 hours after birth, before therapeutic hypothermia.**T1 =** Samples at 3 days. For cooled infants, collected during hypothermia; for controls and non-cooled mild HIE, collected at normothermia (36.5–37.5°C).
GA: Gestational age**, BW:** birth weight

© 2026 Montaldo P et al. *JAMA Network Open.*

eFigure2. Identification of any sources of variance in the dataset.

Two-dimensional principal component analysis (PCA) of mean-centered and scaled whole-genome expression profiles from infants with hypoxic-ischemic encephalopathy (HIE) and healthy controls. Each point represents the genome-expression profile of an individual infant at a given time point. Square markers denote healthy controls, and triangle markers denote HIE infants. Panels depict PCA colored by (**A**) gender, (**B**) gestational age, and (**C**) birthweight. Distinct colors correspond to categories within each variable (male vs. female; gestational-age groups; birth-weight groups).

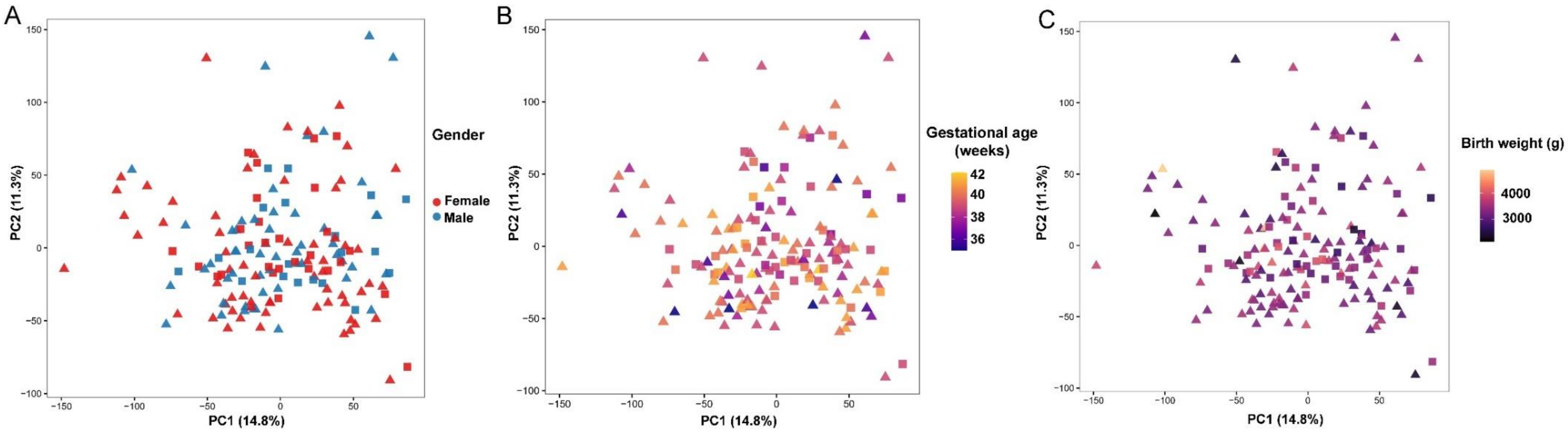

© 2026 Montaldo P et al. *JAMA Network Open.*

eFigure 3. Batch effect correction.

Principal component analysis of batch 1 (Moderate/Severe HIE and matched healthy controls red) and batch 2 (Mild HIE and matched healthy controls green) after quantile normalization and ComBat-Seq. Each dot represents a patient and is colored according to batch number. Square shapes represent moderate/severe HIE, triangle shapes represent mild HIE and circle healthy controls.

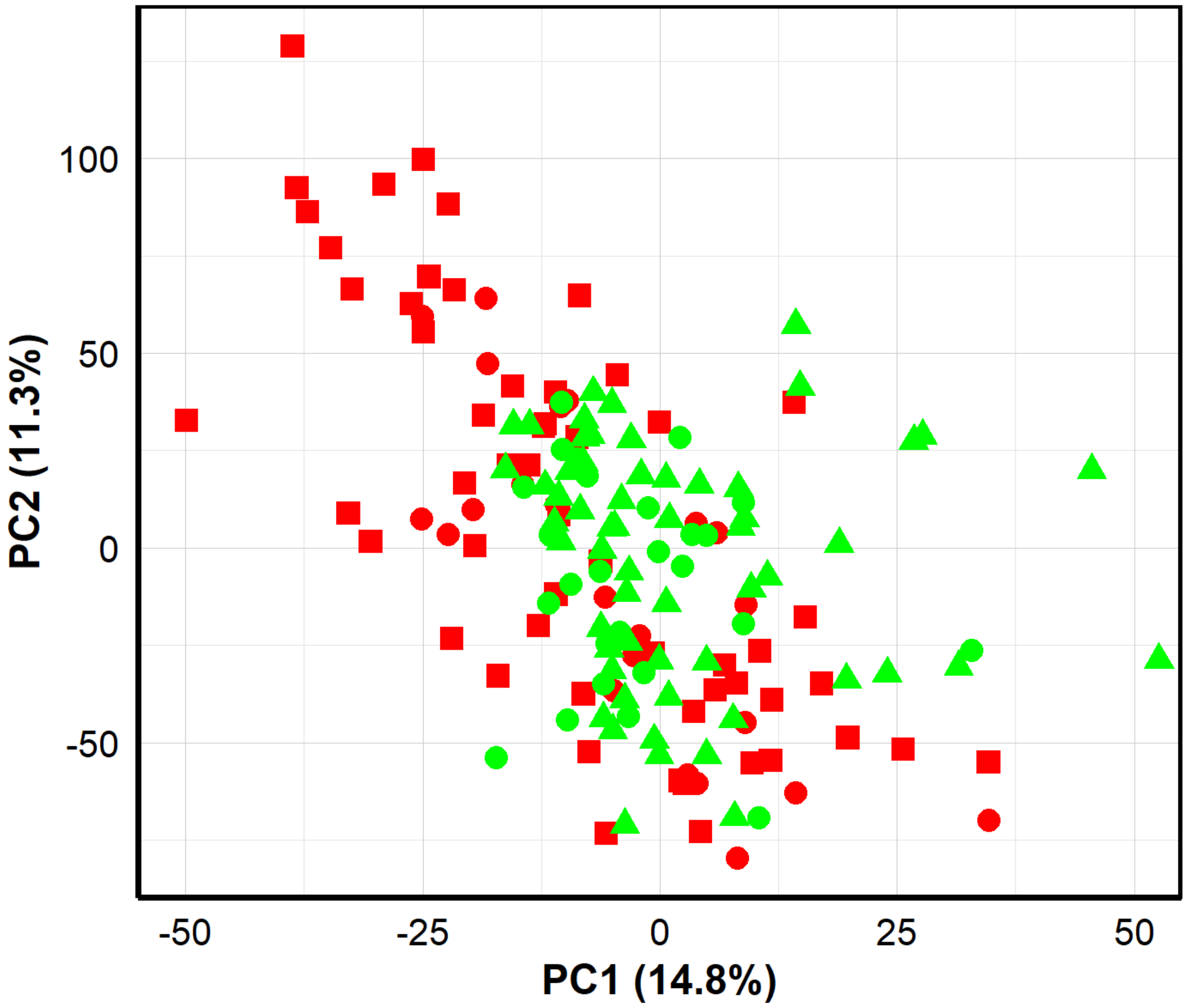

© 2026 Montaldo P et al. *JAMA Network Open.*

eFigure 4. Assessment of variance across HIE severity.

Principal component analysis of whole-transcriptome profiles across infants with mild (blue), moderate (orange), severe (red) hypoxic ischaemic encephalopathy (HIE). Circle shapes represent blood samples collected at birth, triangle shapes blood samples collected at 72 hours.

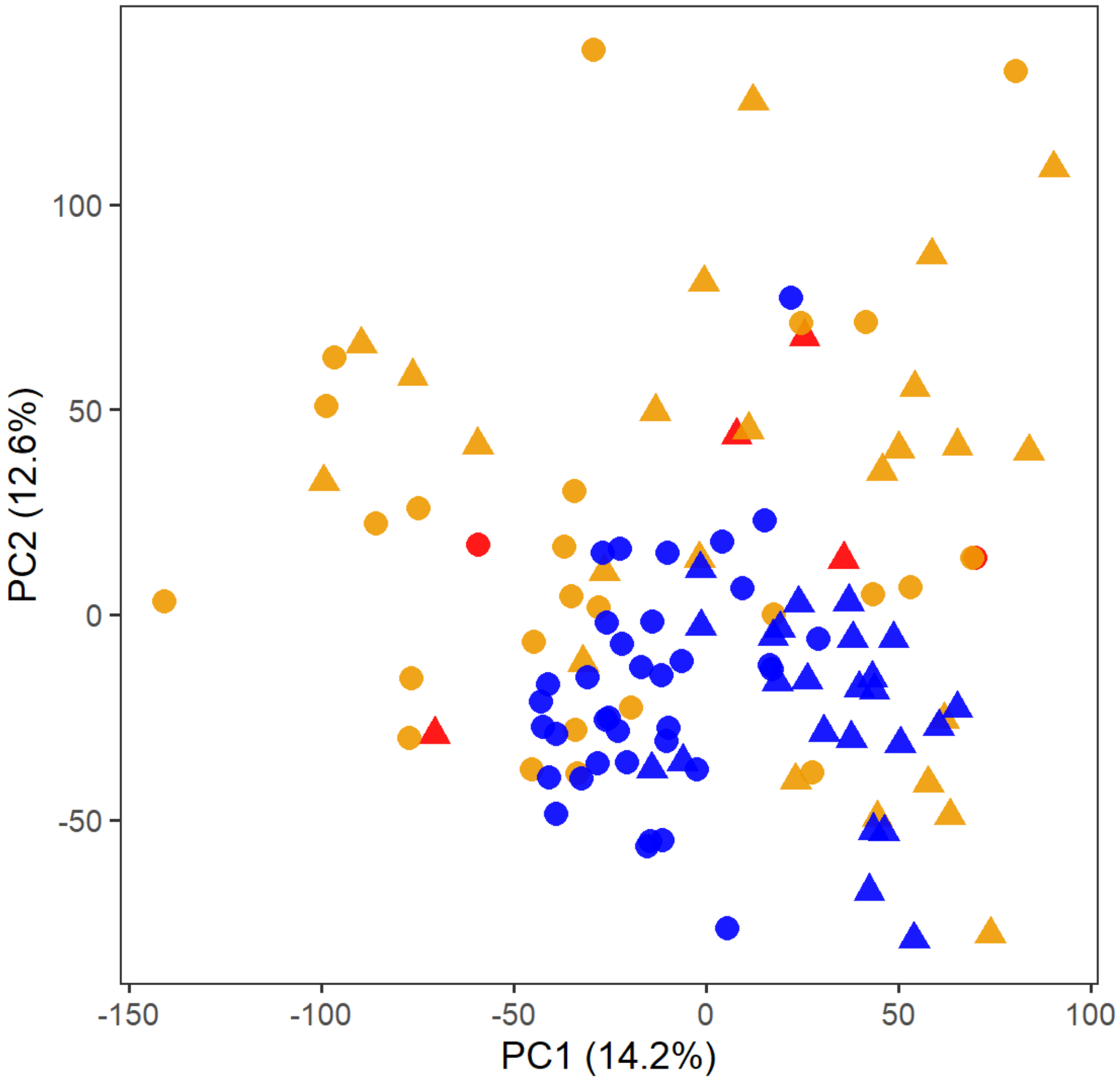

© 2026 Montaldo P et al. *JAMA Network Open.*

eFigure 5. Temporal and severity-dependent gene expression changes in neonatal encephalopathy.

Volcano plots showing differential gene expression between hypoxic ischaemic encephalopathy (HIE) subgroups and healthy controls at two time points (T0 and T1). Each plot displays $\log_2$ fold change (x-axis) versus $-\log_{10}$(p-value) (y-axis), with red dots indicating genes meeting the significance threshold (FDR < 0.05).

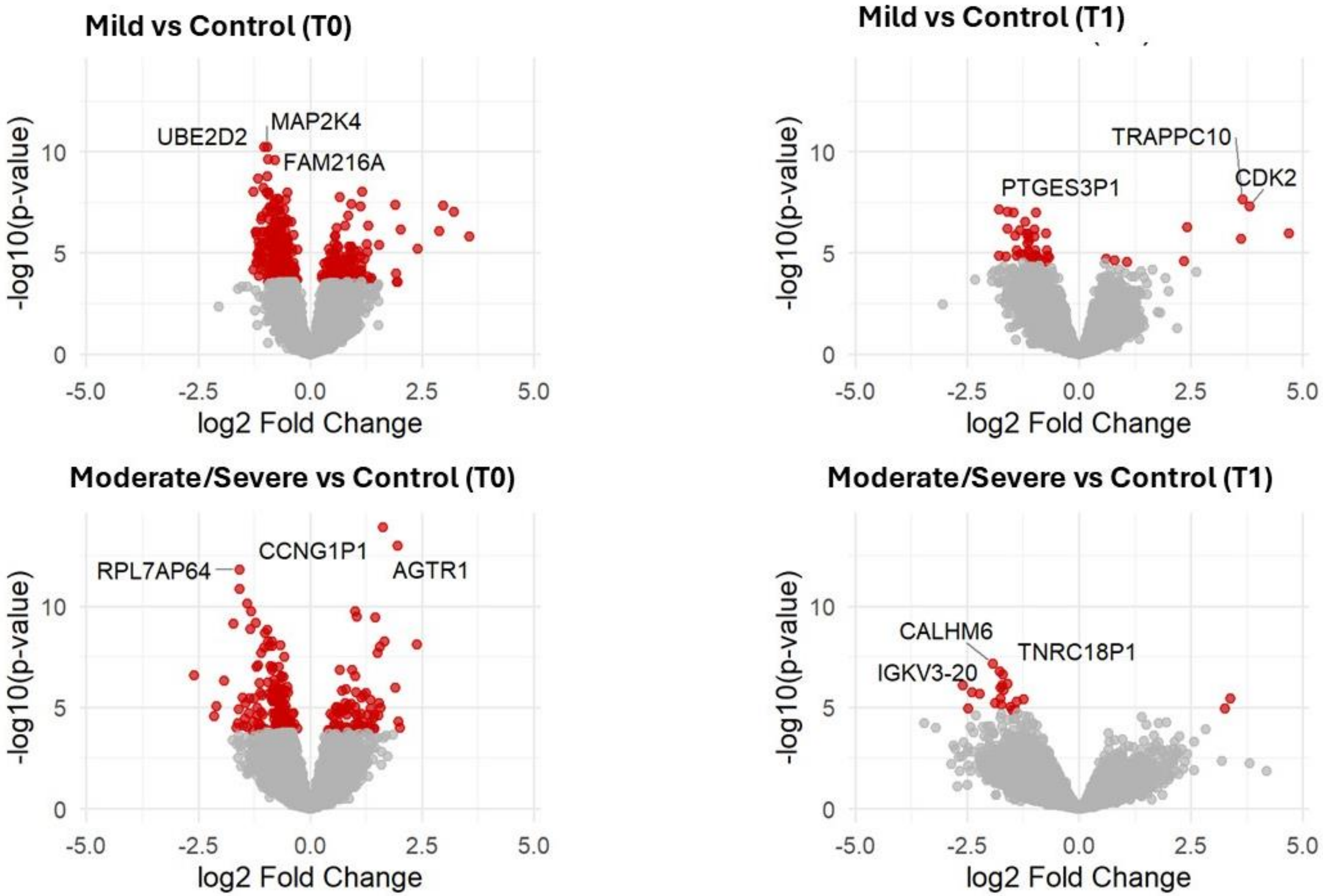

© 2026 Montaldo P et al. *JAMA Network Open.*

eFigure 6. Expression of the top three differentially expressed genes for each pairwise comparison at 72 hours.

Boxplots show log2-transformed, ComBat-corrected expression values for the top three genes in the comparison of infants with mild HIE vs controls (top panel), moderate/severe HIE vs controls (middle panel) and mild vs moderate/severe (bottom panel) comparisons. Each panel displays the distribution of genome expression across the two groups, with individual samples overlaid as points. Boxes represent the interquartile range (IQR), the horizontal line indicates the median, and whiskers extend to 1.5× IQR. Differential expression statistics for the top genes in each comparison: **Mild vs Controls:** TRAPPC10: $\log_2$FC 3.65, CDK2: $\log_2$FC 3.80, PTGES3P1: $\log_2$FC −1.79; all FDR <0.001. **Moderate/Severe vs Controls:** CALHM6: $\log_2$FC −1.92, TNRC18P1: $\log_2$FC −1.76, IGKV3-20: $\log_2$FC −1.69; all FDR <0.001. **Mild vs Moderate/Severe:** LGALS9C: $\log_2$FC 3.82, TREML3P: $\log_2$FC −1.44, FANK1: $\log_2$FC −3.11; all FDR <0.001.

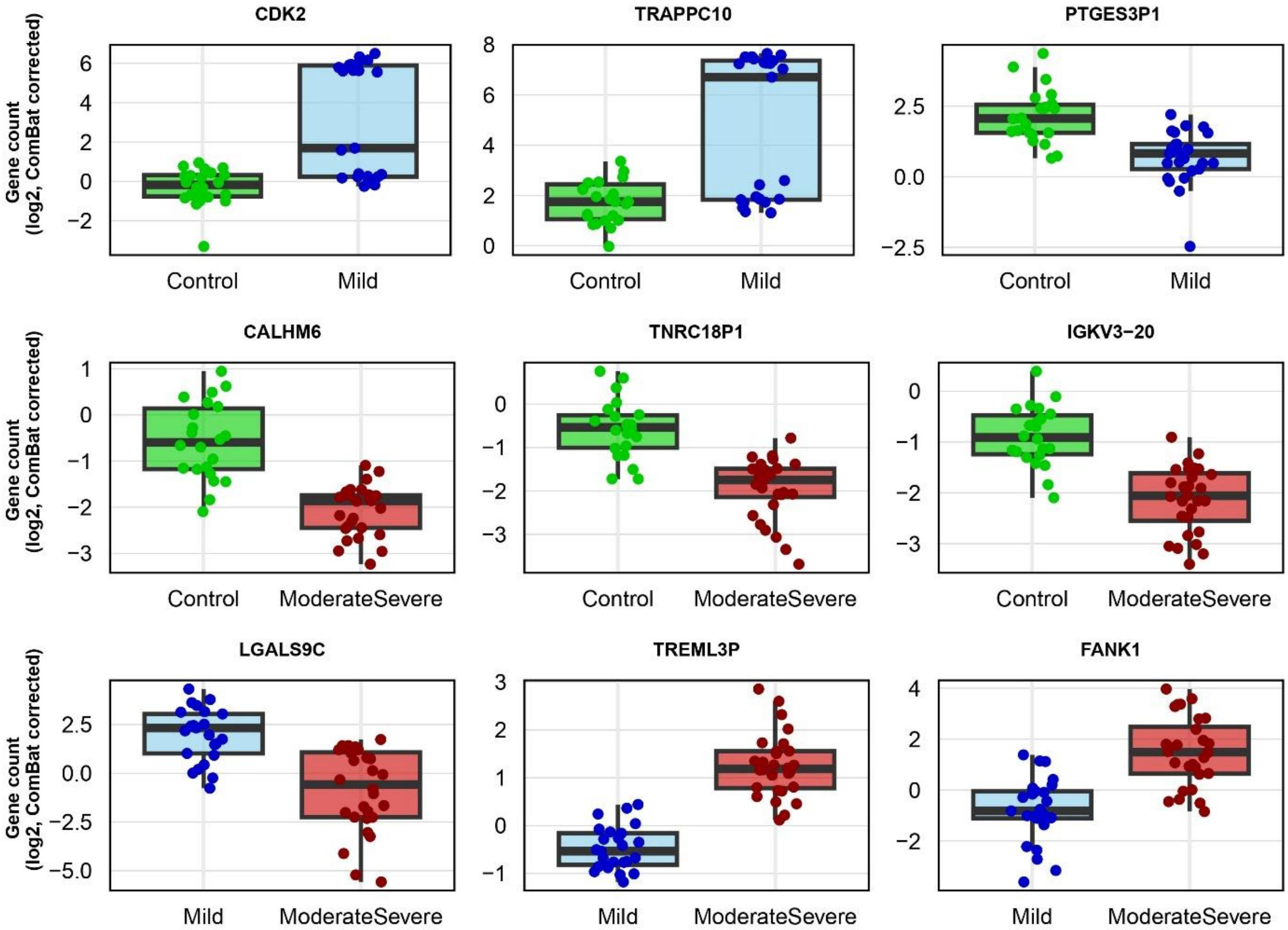

© 2026 Montaldo P et al. *JAMA Network Open.*
