# Supplementary material for: Whole-Blood Gene Expression and Hypoxic-Ischemic Encephalopathy at Birth
Source: JAMA Netw Open. 2026 Aug 21;9(8):e2629378. doi: 10.1001/jamanetworkopen.2026.29378 (PMC13499004; doi:10.1001/jamanetworkopen.2026.29378)
Supplement: Supplementary file 2 [file jamanetwopen-e2629378-s003.pdf]

Data Sharing Statement

Montaldo. Whole-Blood Gene Expression and Hypoxic-Ischemic Encephalopathy at Birth. *JAMA Netw Open*. Published August 21, 2026. doi:10.1001/jamanetworkopen.2026.29378

Data
Data available: No

Additional Information
Explanation for why data not available: Data used the study can be accessed from the published supplementary files.
